# DOCK11 and DENND2A play pivotal roles in the maintenance of hepatitis B virus in host cells

**DOI:** 10.1371/journal.pone.0246313

**Published:** 2021-02-04

**Authors:** Shinichi Hashimoto, Takayoshi Shirasaki, Taro Yamashita, Sadahiro Iwabuchi, Yutaka Suzuki, Yuzuru Takamura, Yoshiaki Ukita, Shungo Deshimaru, Toshitugu Okayama, Kazuho Ikeo, Kazuyuki Kuroki, Kazunori Kawaguchi, Eishiro Mizukoshi, Kouji Matsushima, Masao Honda, Shuichi Kaneko

**Affiliations:** 1 Department of Molecular Pathophysiology, Institute of Advanced Medicine, Wakayama Medical University, Wakayama, Japan; 2 Japan Science and Technology Agency, CREST, Tokyo, Japan; 3 Department of Gastroenterology, Graduate School of Medicine, Kanazawa University, Ishikawa, Japan; 4 Department of Computational Biology, Graduate School of Frontier Sciences, The University of Tokyo, Chiba, Japan; 5 Department of Bioscience and Biotechnology, Japan Advanced Institute of Science and Technology, Ishikawa, Japan; 6 Faculty of Engineering, Graduate Faculty of Interdisciplinary Research, University of Yamanashi, Yamanashi, Japan; 7 Division of Molecular Regulation of Inflammatory and Immune Diseases, Research Institute of Biomedical Sciences, Tokyo University of Science, Noda, Japan; 8 Laboratory of DNA Data Analysis, National Institute of Genetics, Shizuoka, Japan; University of Cincinnati College of Medicine, UNITED STATES

## Abstract

Human hepatitis B virus (HBV) infection remains a serious health problem worldwide. However, the mechanism for the maintenance of HBV in a latent state within host cells remains unclear. Here, using single-cell RNA sequencing analysis, we identified four genes linked to the maintenance of HBV in a liver cell line expressing HBV RNA at a low frequency. These genes included *DOCK11* and *DENND2A*, which encode small GTPase regulators. In primary human hepatocytes infected with HBV, knockdown of these two genes decreased the amount of both HBV DNA and covalently closed circular DNA to below the limit of detection. Our findings reveal a role for *DOCK11* and *DENND2A* in the maintenance of HBV.

## Introduction

It is estimated that 350 million people worldwide are chronic carriers of hepatitis B virus (HBV), which is a principal cause of chronic liver disease. More than 780,000 people die every year due to the complications of hepatitis B infection, including cirrhosis and liver cancer [1]. HBV carriers are much more likely to develop liver cancer than uninfected individuals.

HBV exists as a small, partially double-stranded DNA genome. After infecting hepatocytes, the partially double-stranded genome is converted into covalently closed circular DNA (cccDNA) in the nucleus [2]. HBV can be maintained in a latent state in cells as stably inactivated cccDNA. However, impairment of the immune system by chemotherapy or hematopoietic stem cell transplantation can induce HBV reactivation. In addition, interactions between the host immune system and the virus influence the rate of development of advanced liver disease and hepatocellular carcinoma (HCC). Treatment that successfully reduces viral replication delays the development of advanced liver disease and HCC. However, targeting cccDNA is required for the elimination of latent HBV.

Cells transformed from HBV-infected hepatocytes into HCC cells express reduced amounts of HBV proteins and mRNA intracellularly. Except for cells that contain HBV DNA integrated into the host genome, cell lines established from HCC usually do not express HBV transcripts. Cell lines that continue to express HBV transcripts are very useful for the identification of host factors required for HBV maintenance. Recently, we established an HCC-derived cell line (HC1), in which cccDNA, HBV DNA, and HBV-derived transcripts could be detected. In this study, we performed single-cell transcriptome analysis (or single-cell RNA sequencing, scRNA-seq) [3] to examine the gene expression profile of these cells positive for HBV RNA.

## Materials and methods

### Cell lines

HCC samples were obtained with written informed consent from patients who had undergone radical resection at the Liver Center in Kanazawa University Hospital (Kanazawa, Japan). All patients provided written informed consent. Tissue acquisition procedures were approved by the Ethics Committee of Kanazawa University. Fresh HCC samples were obtained from surgically resected specimens and an autopsy specimen. Primary HCC tissue was dissected and digested in 1 μg/mL type 4 collagenase (Sigma-Aldrich Japan K.K.) solution at 37°C for 15–30 min. Contaminated RBCs were lysed with ammonium chloride solution (STEM CELL Technologies, Vancouver) on ice for 5 min. CD45+ leukocytes and Annexin V+ apoptotic cells were removed using an autoMACS Pro cell separator and magnetic beads (Miltenyi Biotec K.K., Japan). Cells were suspended 1:1 in 200 mL of Dulbecco’s modified Eagle’s medium (DMEM) and Matrigel (BD Biosciences) and subcutaneously injected into six-week-old NOD/SCID mice (NOD.CB17-*Prkdc*^scid^/NCrCrl) purchased from Charles River Laboratories Japan (Yokohama, Japan). When tumors reached 0.5–2.0 cm^3^, the mice were euthanized, followed by digestion and separation of graft tissues as described above. The cells were then seeded in 24-well plates and incubated at 37°C in a humidified atmosphere of 5% CO_2_ for 72 h in DMEM (Gibco BRL, Gaithersburg, MD) containing 10% fetal bovine serum, 1% L-glutamine, and 1% penicillin/streptomycin (normal medium). Non-adherent cells were removed with a pipette, and the culture medium was replaced with normal medium. The adherent cells were maintained in normal medium as an HCC-derived cell line (HC1).

The HC1 genome was sequenced and mapped to the HBV reference genome (Hepatitis B virus, complete genome AJ748098.1) using CLC Genomics Workbench (GWB; QIAGEN, Tokyo, Japan).

### Primary human hepatocyte (PHH) culture and HBV infection

PHHs isolated from chimeric mice with humanized livers were purchased from PhoenixBio Co. (Hiroshima, Japan). PHHs were cultured without passage in 1 mL of DMEM supplemented with 2% FBS, 20 mM HEPES, 44 mM NaHCO3, 15 μg/mL L-proline, 0.25 μg/mL insulin, 50 nM dexamethasone, 5 ng/mL EGF, 0.1 mM Asc-2P and 2% DMSO (2% DMSO-supplemented hepatocyte clonal growth medium [dHCGM]) on type I collagen-coated 12-well plates (2.1 × 10^5^ cells/cm^2^), as described previously [4]. PHHs were infected with HBV particles with 5 GEq/cell, as described previously [5]. PHHs were cultured and infected with HBV under 5% CO_2_ and 95% air at 37°C. Entecavir (ETV: Tokyo Chemical Industry Co, Ltd, Tokyo, Japan) treatment was conducted 1 day after HBV infection. The cells were washed with dHCGM 3 times and on day 2, the cells were washed with dHCGM+ETV (10 nM) 3 times. Briefly, on day 0, a lentiviral particle suspension was added to each well of a plate (PHHs, 7.2 × 10^5^ cells/12 wells). The plate was then covered and transferred to a tissue culture incubator and incubated overnight. On day 1, PHHs were infected with HBV (Genotype C, 5 HBV DNA copies/cell) and washed with buffer. On day 7, the cells were collected to estimate mRNA using SAGE-seq.

### Quantification of HBV DNA and cccDNA using qPCR

HBV DNA was extracted from cells using a DNeasy Blood & Tissue Kit (QIAGEN) according to the manufacturer’s instructions. HBV DNA in the culture medium was extracted using an SMI TEST EX R&D Kit (MLB, Nagoya, Japan) according to the manufacturer’s instructions. DNA was quantified via qPCR analysis using the primer set 5’-ACTCACCAACCTCCTGTCCT-3’ and 5’-GACAAACGGGCAACATACCT-3’ and probe 5’-FAM/TATCGCTGG/ZEN/ATGTGTCTGCGGCGT/3IBFQ. To isolate cccDNA, the extracted DNA (50 ng) was treated for 60 min at 37°C with 10U Plasmid safe DNase I (Epicentre, Madison, WI) and then treated for 30 min at 70°C for DNase inactivation. cccDNA (2.5 ng) was quantified via qPCR using the primer set 5’-CGTCTGTGCCTTCTCATCTGC-3’ and 5’-GCACAGCTTGGAGGCTTGAA-3’ and probe 5’-FAM-CTGTAGGCATAAATTGGT-MGB-3’ [2]. For Southern blotting, cccDNA was extracted from HBV-infected cells using the KCl protein precipitation method, separated through a 0.8% agarose gel, blotted onto a nylon membrane, and hybridized with a 32P HBV DNA probe.

### Knockdown of *DENND2A*, *LIPG*, *DOCK11*, *HECW2*, and *CDC42* using shRNA

The roles of *DENND2A*, *DOCK11*, and *CDC42* in viral maintenance were examined using shRNA (S1 Table) (MISSION®, Sigma, St. Louis, MO) in HepG2.2.15 cells and PHHs. Each target gene was cloned into a lentivirus-based pLKO.1-puro shRNA expression vector. For each gene, a target set consisting of 4–5 individual constructs was prepared, targeting different regions of the gene sequence. In addition to the viruses generated for the human genes, negative mock controls (empty vector and non-target shRNA controls) were prepared to monitor transduction efficiency and to enable a well-to-well comparison of results. For HepG2.2.15 cells, on day 1, the lentiviral particle suspension was added to each well of the plate. The plate was incubated overnight at 37°C. On day 2, the plates were washed with buffer. From days 3–6, the culture medium was replaced as necessary. On day 6, the cells were collected for the measurement of HBV content. For PHHs, on day 0, the lentiviral particle suspension was added to each well of a plate containing PHHs (7.2 x 10^5^ cells/12 wells). The plate was incubated overnight at 37°C. On day 1, PHHs were washed and infected with HBV (Genotype C, 5 HBV DNA copies/cell). On day 2, plates were washed with buffer, and 100 mL of complete medium containing puromycin (1 μg/mL) was added to each well. ETV (10 nM; Tokyo Chemical Industry Co, Ltd) treatment was conducted one day after HBV infection. From days 3–6, the culture medium was replaced as necessary. On day 6, the cells were collected for the measurement of HBV content.

### Overexpression of *DOCK11 and CDC42*

The Halo-tagged DOCK11 cDNA plasmid was purchased from the Kazusa DNA Research Institute (Chiba, Japan). CDC42 and DOCK11 expression plasmids were transfected into HepG2.2.15 cells using the Lipofectamine 3000 Transfection Reagent according to the manufacturer’s instructions.

### Anti-hepatitis C virus (HCV) activity of *DOCK11* shRNA

Anti-HCV activity of *DOCK11* shRNA was evaluated using a JFH1 replicon system. HCV replication analysis was performed by transfecting Huh7.5 cells with synthetic JFH1-RNA. Huh7.5 cells were transfected with 1 μg synthetic RNA with the TransIT^®^-mRNA Transfection Kit (Takara, Japan. At 24, 48, and 72 h after transfection, total RNA was isolated using a High Pure RNA Isolation Kit (Roche Diagnostics, GmbH, Germany), and cDNA was synthesized using a high-capacity cDNA reverse transcription kit (Applied Biosystems, Carlsbad, CA). HCV RNA was measured as described previously [6].

### Statistical analysis

Pairwise comparisons were performed using Student’s t-tests (two-tailed). Multiple-group comparisons were performed using one-way analysis of variance with Bonferroni’s post-hoc test. Data are presented as the mean ± SD. *P* <0.05 was considered to indicate a statistically significant difference.

### Accession numbers

Nx1-seq data have been deposited in the DDBJ DNA Databank of Japan with accession number DRA005282.

## Results

### Establishment of an HCC cell line

**Fig 1:**
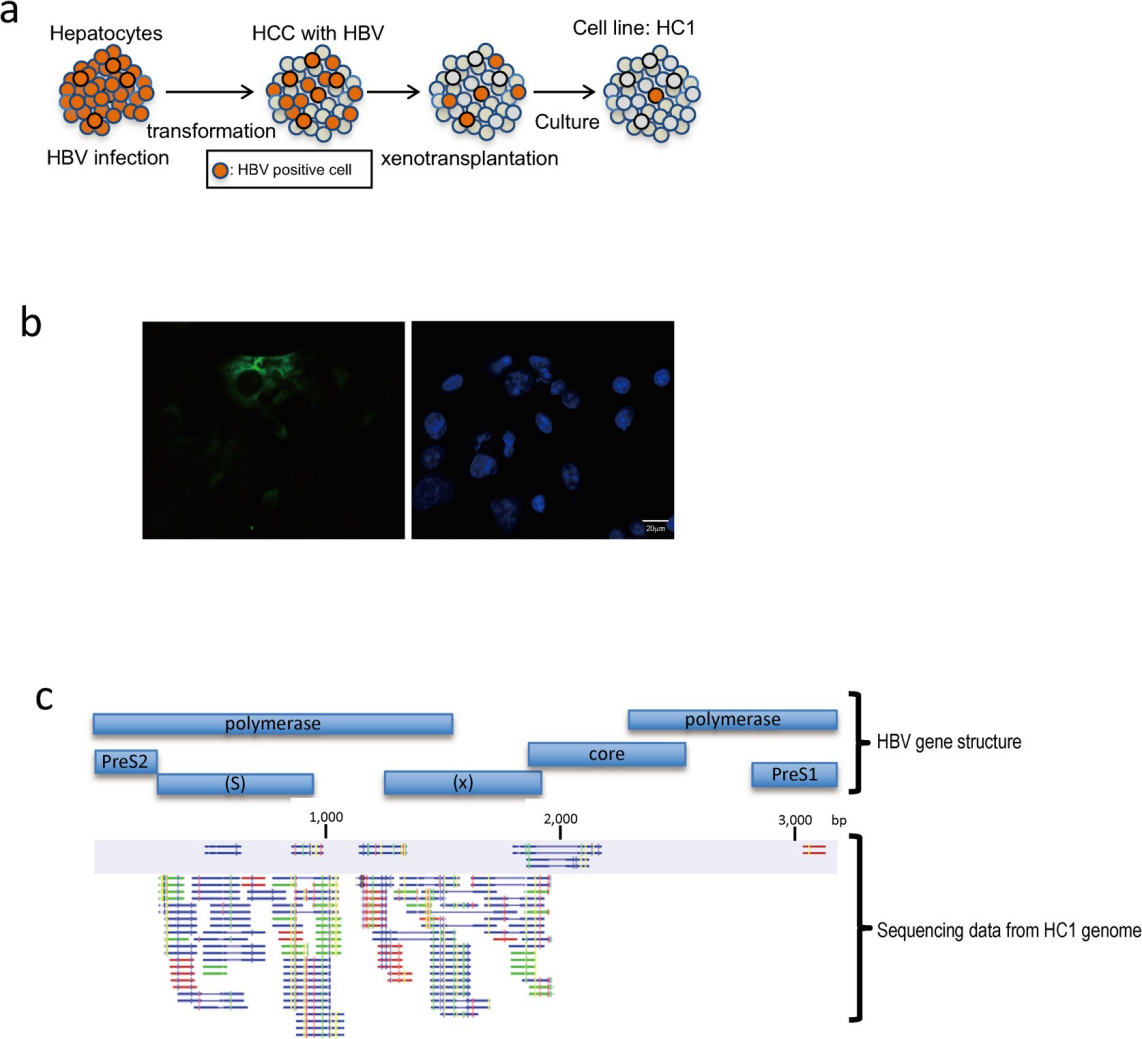
Cell lines continually expressing HBV transcripts. (a) Conceptual diagram illustrating the decreasing number of HBV-positive cells during transformation and culture of hepatocytes. (b) Representative image of immunostaining results of hepatitis B core antigen (HBc) in HC1 cells (left). Cell nuclei are stained with DAPI (right). (c) Mapping results showing high specificity to the HBV genome integrated into the HC1 genome. Not all components of the HBV genome were integrated into the host HC1 genome. HC1 genome was sequenced and was mapped to the HBV reference genome as described in Materials and methods. The sequencing was performed twice, and the first result is shown as a light purple band. The blue bars indicate the gene position that codes each of the transcripts in the HBV genome. Each color of small lines show the mapped DNA derived from the HC1 genome. CLC GWB was used as a read mapping tool. Read colors: blue, green, red, and yellow indicate pair read, forward, complementary, and nonspecific, respectively.

Fresh HCC samples were obtained from surgically resected specimens and an autopsy specimen and were used immediately for the preparation of single-cell suspensions and xenotransplantation. Fig 1A shows the process by which hepatocytes acquire the properties of cancer (transformation) and cell lines are prepared from the cells by transplanting live cancer cells into individual mice (xenotransplantation). We then established an HCC-derived cell line (HC1) in which we could detect cccDNA and HBV DNA as well as HBV-derived transcripts. Fig 1B shows the immunostaining of hepatitis B core antigen (HBc) in HC1 cells, wherein less than 0.1% of cells were positive for the staining. As HBV DNA integrants in the host chromosomes are often rearranged or duplicated [7], it is unclear whether HBV in the cells could indeed be distinguished between integrated HBV DNA and cccDNA. The possibility that HBV mRNA was generated from integrated HBV DNA cannot be ruled out. Moreover, due to the lack of selection pressure, the passages of HC1 cells may select for rapidly proliferating cells and lead to the loss of HBV-positive cells. Therefore, to prove that these genes and proteins were not derived from the inserted gene, the genomic DNA of HC1 was sequenced. As shown in Fig 1C, not all components of the HBV genome were integrated into the host HC1 genome. As a result, it was found that the HBV-derived proteins and genes were not derived from HBV genes inserted into the genome. These data indicated that the HC1 cells were infected by HBV. We speculated that these positive cells might possess a mechanism for maintaining HBV. Therefore, using these cells, we performed scRNA-seq analysis to identify HBV maintenance-related genes from HBV-positive HC1 cells.

### scRNA-seq analysis of the HC1 cell line

We performed well-based scRNA-seq (Nx1-seq, S1A Fig) on 2,325 single cells from HC1 cells with over 1 × 10^5^ sequenced reads per cell. The greatest number of reads obtained from a single cell was approximately 6.6 × 10^6^, which identified approximately 1.2 × 10^3^ different genes (S1B Fig and S2 Table). The total sequenced tags from all 2,325 cells identified approximately 1.9 × 10^3^ different genes.

### Identification of host factors required for the maintenance of HBV DNA and cccDNA

To identify the genes required for the maintenance of HBV in HC1 cells, we first aligned single-cell expression data to the HBV genome. Among the 2,325 cells analyzed, only two cells expressed HBV mRNA, one to a much higher extent than the other (S2 Table). This result is consistent with data showing that a few HC1^HBV^ cells stained positive for HBc using a specific antibody.

To identify genes specifically expressed in HC1^HBV^ cells, we compared the gene expression profiles of the two HC1^HBV^ cells with those of the other 2,323 HBV-free HC1 cells (S3 Table). We limited our analysis to genes expressed in less than 20 cells per 2,325 cells, which were expressed in more than 100 tags per single-cell library. Using these criteria, no genes common to both HC1^HBV^ cells were found. However, when we analyzed only HC1^HBV^ cells with high HBV expression, we identified five genes that were expressed at higher levels in the HC1^HBV^ cells than in HBV-free HC1 cells (S4 Table). Among the proteins encoded by the genes in this set that could be recognized by specific antibodies, confocal microscopy indicated colocalization of DOCK11 and DENND2A with the HBV-associated protein HBc in HC1^HBV^ cells (S2 Fig and Table 1).

**Table 1:** Highly expressed genes in HBV-positive cells.

Gene	Frequency per 2,325 cells*	Tag counts of HBV-positive cells (/million)	Official full name	Refseq	Gene Ontology function
*DENND2A*	7	218	DENN/MADD domain containing 2A	NM_015689	Rab guanyl-nucleotide exchange factor activity
*DOCK11*	16	103	Dedicator of cytokinesis 11	NM_144658	Rho GTPase binding, Rho guanyl-nucleotide exchange factor activity, protein binding

* Number of cells associated with each gene.

### Knockdown of *DOCK11* and *DENND2A* using shRNA

To determine the influence of the genes *DOCK11* and *DENND2A* on HBV replication and maintenance, we used targeted shRNAs to knock down each gene in HepG2.2.15 cells, which secreted complete HBV particles. Five shRNAs for *DOCK11* and four shRNAs for *DENND2A* were used to account for off-target effects and toxicity. Most of the shRNAs significantly reduced the expression of these two genes (S3 Fig). In addition, knockdown of these genes reduced HBV DNA and cccDNA content (S4 Fig) in HepG2.2.15 cells. However, the discrepancy between gene knockdown efficacy and impact on HBV replication was observed in several *DOCK11* shRNA-transfected cells. The reason for the discrepancy between knockdown efficiency of *DOCK11* and HBV-DNA/cccDNA reduction for some *DOCK11* shRNAs is that the shRNA sequences may have affected other host genes besides *DOCK11*.

### Knockdown of *DOCK11* or *DENND2A* reduces HBV DNA and cccDNA content in PHHs

**Fig 2:**
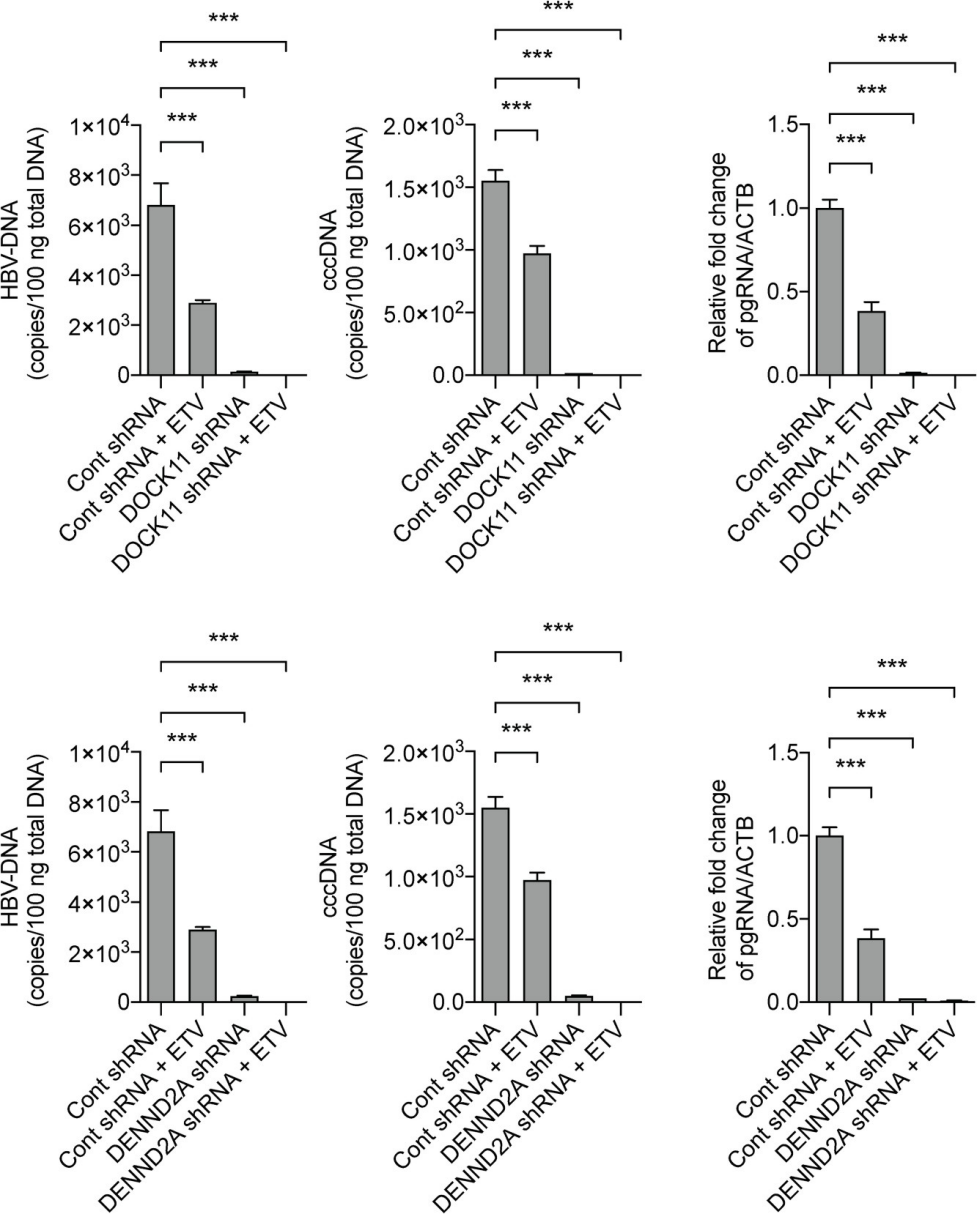
shRNA knockdown of *DOCK11* and *DENND2A* in PHHs reduces HBV DNA and cccDNA. PHH were treated with shRNA targeting *DOCK11* or *DENND2A*, or a combination of the shRNA and ETV. HBV DNA, pregenomic RNA (pgRNA), and cccDNA were measured using qPCR. In this experiment, *DOCK11* shRNA#2 and *DENND2A* shRNA#2 were used. ACTB; actin beta. Data represent mean ± SD pooled from three independent experiments. HBV DNA, pregenomic RNA, and cccDNA contents are compared to that of mock-transfected controls (cont shRNA). *P < 0.05; **P < 0.01; ***P < 0.001.

We isolated PHHs from chimeric mice with humanized livers and infected these cells with HBV. Five days following HBV infection, transfection with shRNA targeting the GTPase-related genes *DENND2A* and *DOCK11* reduced HBV DNA to 2% and 3%, and cccDNA to 1% and 3% of the mock-transfected controls, respectively (Fig 2). In addition, none of the shRNA treatments induced toxicity in PHHs over a six-day observation period.

It is thought that pregenomic HBV RNA is maintained intracellularly after HBV infection. To examine the influence of these genes on the persistence of HBV in PHHs, we co-treated cells with the antiretroviral medication ETV (Fig 2). When combined with ETV treatment, ETV and *DOCK11-* or *DENND2A*-targeted shRNA treatment synergistically reduced HBV DNA and cccDNA to below the limit of detection. These findings suggest that the mechanism by which gene knockdown reduces HBV DNA and cccDNA levels is likely to be independent of the mechanism responsible for the inhibition of reverse transcription during viral replication. However, the underlying mechanism remains poorly understood, and we would like to explore this in a future study.

### Knockdown of *DOCK11* and *DENND2A* during long-term culture reduces cccDNA and pregenomic RNA to below the limit of detection in PHHs

**Fig 3:**
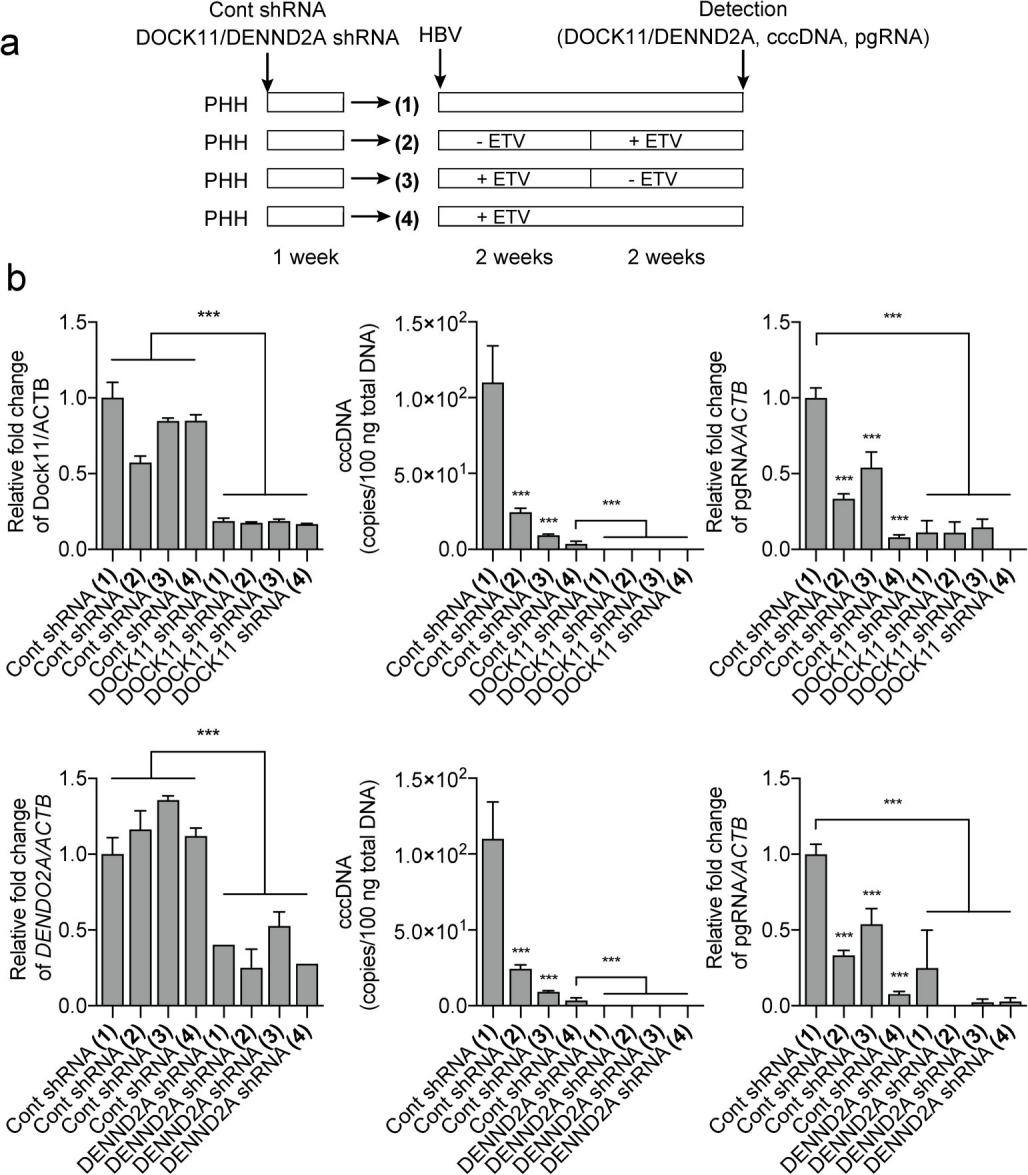
shRNA against *DOCK11* and *DENND2A* reduced cccDNA in PHH during long-term culture. (a) Experimental schedule. PHHs were transfected with shRNA one day before infection with HBV and then cultured for two or four weeks with regular exchange of the culture media, in the presence or absence of ETV. (b) After HBV infection, shRNA targeted to *DOCK11* reduced cccDNA and pregenomic RNA (pgRNA) levels. DNA and RNA extracts from infected cells were treated as indicated. Data represent mean ± SD pooled from three independent experiments. *P < 0.05; **P < 0.01; ***P < 0.001.

We next examined the effect of knockdown of *DOCK11* and *DENND2A* on HBV DNA and cccDNA levels in long-term cultures of PHHs after HBV infection. PHHs were transfected with shRNA one day before infection with HBV and then cultured for four weeks with regular exchange of the culture media in the presence or absence of ETV (Fig 3A). Knockdown of *DENND2A* for a month reduced the survival of PHHs to 60%, while knockdown of *DOCK11* did not affect survival (S5 Fig). In long-term culture, *DOCK11* and *DENND2A* knockdown reduced cccDNA and pregenomic RNA content to below the limit of detection (141 copies/2.5 ng DNA), even in the absence of ETV (Fig 3B).

#### Knockout of *DOCK11* and *DENND2A* using the CRISPR/Cas9 system

We next used the CRISPR/Cas9 system to knock out *DOCK11* and *DENND2A* gene expression, since shRNA sometimes has nonspecific effects at both the mRNA and protein levels, such as off-target mRNA degradation, protein regulation, and induction of the interferon (IFN) response (S6 Fig). Knockout of *DOCK11* reduced cccDNA content to 5% of mock-transfected control levels in HepG2.2.15 cells without cell death, indicating that *DOCK11* plays an important role in the maintenance of cccDNA. In contrast, the use of CRISPR/Cas9 system to knockout *DENND2A* induced cell death up to 50% of the levels observed in mock-transfected controls. Therefore, we next focused on the effects of *DOCK11* knockout.

#### Knockdown of *DOCK11* eliminates established HBV infection

**Fig 4:**
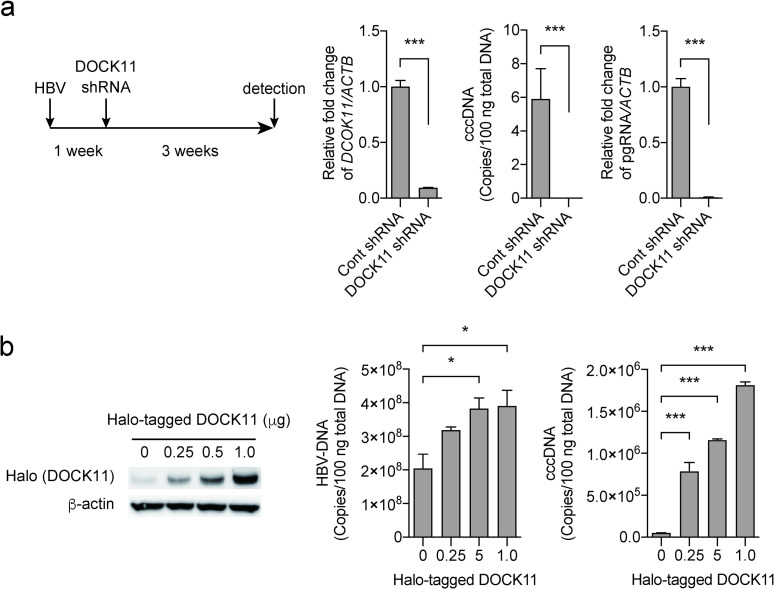
Effect of knockdown and overexpression of *DOCK11* in HBV-infected and HepG2.2.15 cells. (a) The schematic represents the infection/treatment regimen and its duration. Subsequently, cccDNA and pregenomic RNA (pgRNA) were measured using real time PCR. (b) Halo-tagged DOCK11 plasmid was transfected into HepG2.2.15 cells and the HBV DNA and cccDNA levels were quantified 72 h after transfection. Left figure of western blotting shows the correlation between the halo tag and DOCK11. Data represent mean ± SD pooled from three independent experiments. *P < 0.05; **P < 0.01; ***P < 0.001.

We have described above the inhibition of cccDNA formation in PHHs infected with HBV after treatment with shRNA. Further, we examined the effects of *DOCK11* shRNAs on PHHs in which HBV infection was already established. When PHHs were infected with HBV, followed by treatment with *DOCK11*-targeting shRNA one week later, and then cultured for a further three weeks with regular exchange of culture media, cccDNA and pregenomic RNA completely disappeared without cell death (Fig 4A). To further determine if *DOCK11* is important for HBV maintenance, we prepared *DOCK11*-overexpressing cells. As shown in Fig 4B, we transfected the Halo-tagged *DOCK11* plasmid into HepG2.2.15 cells and quantified HBV DNA and cccDNA levels 72 h after transfection. *DOCK11* overexpression clearly increased HBV DNA and cccDNA levels. Taken together, these findings suggest that the *DOCK11* gene product is involved in the maintenance of the HBV genome.

#### Knockdown of the DOCK11-associated protein CDC42 reduces HBV cccDNA

**Fig 5:**
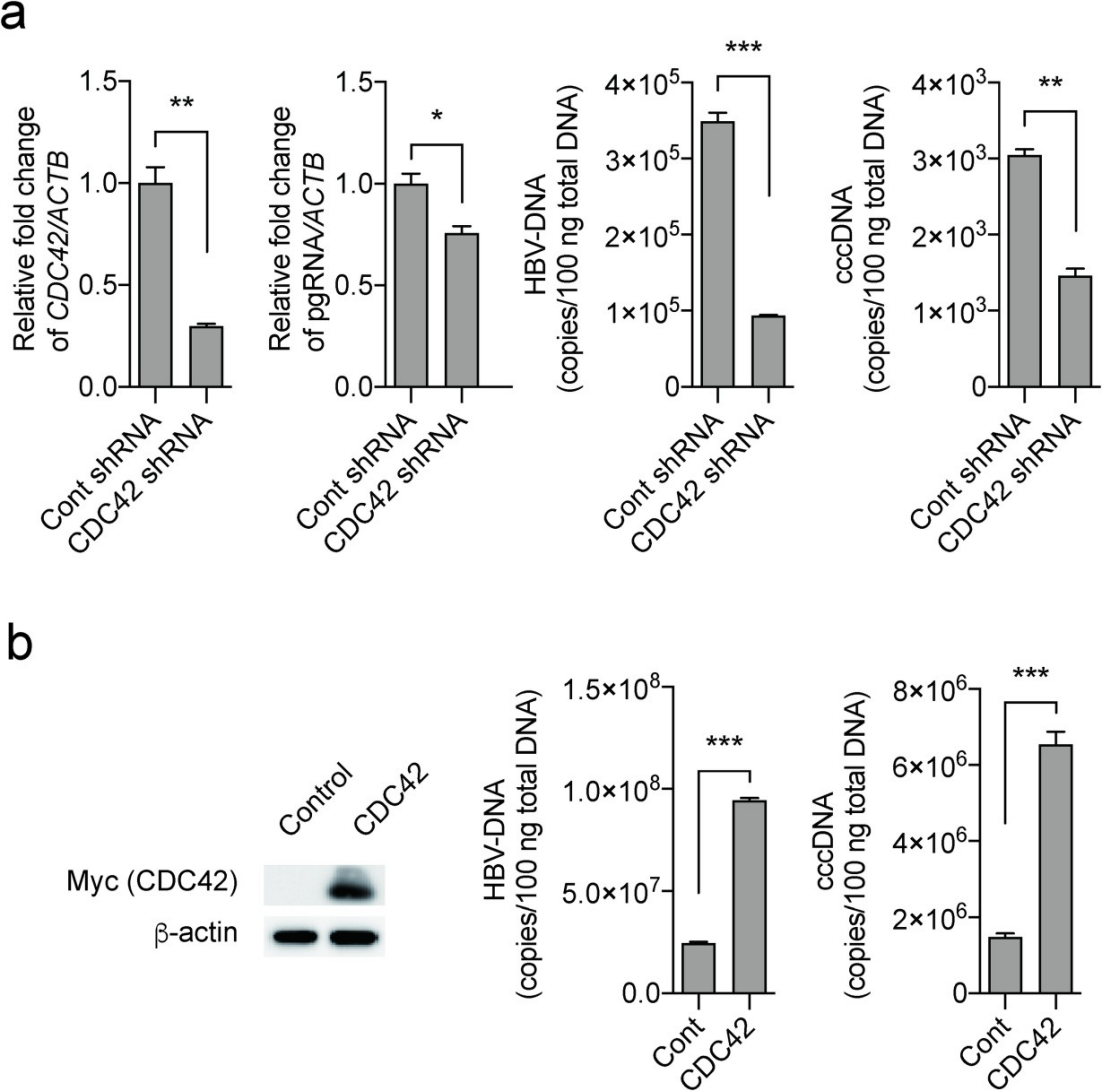
Effect of knockdown and overexpression of *CDC42 in HBV-infected and HepG2*.*2*.*15* cells. (a) shRNA targeting *CDC42* reduced HBV DNA, cccDNA, and pregenomic RNA (pgRNA) levels in HepG2.2.15. (b) The Myc-tagged CDC42 plasmid was transfected into HepG2.2.15 cells, and HBV-DNA and cccDNA levels were quantified 72 h after transfection. Left figure of western blotting shows the correlation between Myc-tagged CDC42 and DOCK11. cDNA plasmid was purchased from OriGene (Rockville, MD). Data represent mean ± SD pooled from three independent experiments. *P < 0.05; **P < 0.01; ***P < 0.001.

DOCK11 is an exchange factor for the Rho GTPases RAC and CDC42, and overexpression of DOCK11 activates CDC42 [8]. We thus investigated the effect of *CDC42* knockdown on HBV DNA and cccDNA levels in HepG2.2.15 cells (Fig 5A). Knockdown of *CDC42* reduced HBV DNA and cccDNA contents to less than 50% of mock-transfected control levels in HepG2.2.15 cells. Moreover, to confirm the relationship between *CDC42* and HBV content in infected cells, HepG2.2.15 cells overexpressing *CDC42* were prepared. As shown in Fig 5B, Myc-tagged CDC42 plasmid was transfected into HepG2.2.15 cells, and HBV-DNA and cccDNA levels were quantified 72 h after transfection. CDC42 overexpression clearly increased HBV DNA and cccDNA levels. Thus, CDC42 is important for HBV maintenance, and DOCK11 may mediate the maintenance of cccDNA, at least in part, via the CDC42 pathway.

#### *DOCK11* knockdown reduces cellular HCV content

**Fig 6:**
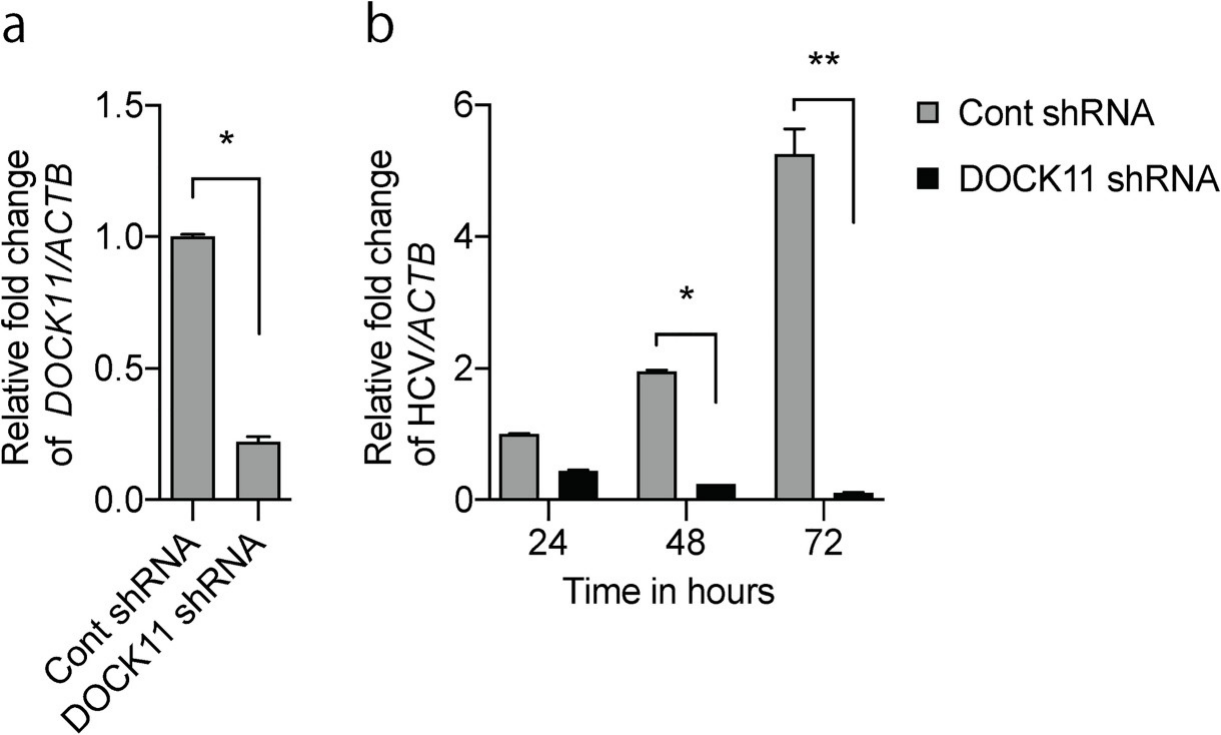
Role of *DOCK11* in HCV replication. A Gaussia luciferase assay was performed on human hepatocytoma Huh7.5 cells infected with HCV. In *DOCK11* shRNA-treated Huh7.5 cells, a significant reduction in *DOCK11* expression was confirmed via real-time PCR (a). *DOCK11* knockdown reduced cellular HCV content 24, 48, and 72 h after synthetic HCV RNA transfection (b). shRNA targeting *DOCK11* reduced virus replication in Huh7.5 cells infected with HCV. At days 1–7 after transfection, cells were harvested to assay their luciferase activity. Data represent mean ± SD pooled from three independent experiments. *P < 0.05; **P < 0.01; ***P < 0.001.

RAC GTPase-activating protein 1 (RACGAP1) plays an important role in the hydrolysis of GTP to GDP with RAC1 and CDC42, raising the possibility that DOCK11 and RACGAP1 share downstream effectors. In addition, it has been reported that siRNA-mediated knockdown of RACGAP1 in human hepatoma cell lines inhibits the replication of HCV RNA [9]. To examine the role of DOCK11 in HCV replication, we performed a Gaussia luciferase assay on human hepatocytoma Huh7.5 cells infected with HCV. In *DOCK11* shRNA-treated Huh7.5 cells, a significant reduction in *DOCK11* expression was confirmed using real-time PCR (Fig 6A). *DOCK11* knockdown reduced cellular HCV content 24, 48, and 72 h after synthetic HCV RNA transfection (Fig 6B). These results indicate that *DOCK11* knockdown reduces HCV replication and suggests a role for DOCK11 in the lifecycle of other viruses in addition to HBV.

## Discussion

Unlike previous studies that have sought to identify HBV-related genes involved in the life cycle of the virus, this study was performed to identify specific cellular components required for the maintenance of HBV in host cells. We utilized Nx1-Seq single-cell transcriptome analysis to investigate gene expression in a small minority of cells in the newly established HC1 cell line that maintained HBV expression during culture. The decrease in HBV RNA that was observed as HC1 cells were passaged parallels the decrease in the number of HBV-positive cells observed during the transition from HBV-infected hepatocytes to cancer cells and from cancer cells to established cell lines. We hypothesized that this decrease in HBV expression may result from the deletion, mutation, and/or epigenetic changes in genes involved in HBV maintenance. We thus used Nx1-Seq to identify genes and proteins whose expression was specifically retained in the cells maintaining HBV expression. This analysis identified *DOCK11* and *DENND2A* as candidate genes required for the intracellular maintenance of HBV.

In PHHs infected with HBV, knockdown of *DOCK11* and *DENND2A* strongly decreased the amount of both HBV DNA and cccDNA to below the limit of detection, indicating an essential role of these genes in the maintenance of HBV in host cells. In addition, these genes were not induced in PHHs or cell lines by HBV infection. The reduction of DOCK11 and DENND2A levels by using shRNA is mechanistically linked to inhibition of HBV replication. This could be mediated by off-target effects and/or on- or off-target toxicity. shRNA sometimes has nonspecific effects at both the mRNA and protein levels. shRNA treatment in combination with ETV promotes synergistic reduction of HBV replication through independent mechanisms, such as metabolism of HBV. To clarify this point, we would like to explore this in a future study.

Our data showed that knockdown of *CDC42* reduced HBV DNA and cccDNA content to less than 50% of mock-transfected control levels in HepG2.2.15 cells. Moreover, to further determine whether CDC42 is crucial for HBV maintenance, we generated CDC42-overexpressing cells. We transfected Myc-tagged CDC42 plasmid into HepG2.2.15 cells and quantified HBV DNA and cccDNA levels 72 h after transfection. CDC42 overexpression clearly increased HBV DNA and cccDNA levels. The Rho family of small GTP-binding proteins—CDC42, RAC1, and RHOA—regulates diverse cellular functions such as adhesion, polarization, spreading, migration, endocytosis, and cell growth [10]. Tan *et al*. [11] reported that activated RAC1 and CDC42 increase HBV replication, whereas a dominant negative form of RAC1 reduces HBV replication. Our findings suggest that DOCK11 is likely to work downstream of CDC42 and RAC1 to induce changes in the actin cytoskeleton and HBV replication. The DOCK11-associated protein CDC42 is a key moderator of cellular actin dynamics, and is important for cell entry and trafficking for numerous viruses. Thus, the DOCK11-CDC42 signaling pathway may be essential in the viral life cycle or viral infections.

Recently, Lucifora *et al*. [12] showed that IFN upregulates APOBEC3A and APOBEC3B cytidine deaminases in HBV-infected cells, resulting in the degradation of nuclear viral DNA. Similarly, it has previously been shown that APOBEC3G inhibits the packaging of pregenomic RNA in the HBV capsid and thus promotes the clearance of HBV DNA in a non-cytolytic manner [13,14]. In addition, combination treatment with IFN-alpha and the nucleotide analogue ETV reduced cccDNA levels. However, when we knocked down *DOCK11* in HBV-infected PHHs, there was no change in *APOBEC3G* or *APOBEC3A* expression. Thus, *DOCK11* knockdown is likely to reduce cccDNA content via an alternative pathway.

DENND2A is a member of the DENN domain family that localizes to actin filaments. DENN domain-containing proteins interact directly with members of the Rab family of small GTPases, and the DENN domains function enzymatically as Rab-specific guanine nucleotide exchange factors [15]. Interestingly, it was recently shown that the Rho family protein RHOBTB3 is a RAB9 effector protein [16]. Rho family proteins are typically associated with processes controlling the actin cytoskeleton. In addition, it has been reported that DENND2 family members act as guanine nucleotide exchange factors for RAB9 trafficking between late endosomes and the trans-Golgi network. Components of the BLOC complex involved in trafficking to lysosome-like organelles have been found to be associated with actin filaments [17] and to interact with RAB9 [18]. Yoshimura *et al*. have reported that DENND2 GDP-GTP exchange factors target actin filaments and control RAB9-dependent trafficking of mannose-6-phosphate receptors to lysosomes [19]. Although the relationship between HBV DNA, cccDNA, and DENND2A remains unclear, knockdown of DENND2A may inhibit RAB9-mediated HBV trafficking, which is an essential stage in the viral life cycle.

A common property of *DOCK11* and *DENND2A* may be associated with cytoskeleton-related proteins, such as actin filaments or tubulin that are used to transport HBV DNA into the nucleus or to stabilize cccDNA within the nucleus. Using the VaPros and STRING databases, we investigated the pathways involving *DOCK11*-related genes before and after shRNA treatment. A KEGG pathway of 13 genes associated with DOCK11 revealed an association with “Focal adhesion”, “Regulation of actin cytoskeleton”, “Bacterial invasion of epithelial cells”, and “Shigellosis” as four areas in which the generation of the cytoskeletal protein actin is suppressed. This finding suggests that cytoskeleton-related genes may be very important for the maintenance of HBV in host cells. However, this study only conducted *in vitro* experiments, and the effects in an *in vivo* setup are unknown. Therefore, we plan to investigate the effects of this gene using liver-humanized mice in future studies. In addition, we must investigate the role of DOCK11 and DENND2A in vivo to promote therapeutic strategy against HBV infection.

## Conclusions

In conclusion, we have shown that *DOCK11* and *DENND2A* are essential for the maintenance of HBV in infected cells. Given that *Dock11* does not play a critical role in the regulation of viability, fertility, or development in mice [20], molecules inhibiting DOCK11 could be an effective therapeutic strategy to combat HBV. These findings will greatly contribute to the development of HBV biology.

## Supporting information

S1 Fig scRNA-seq analysis of the HC1 cell line. (a) Outline of the Nx1-Seq approach. First, single-cell and oligo-dT barcode beads are combined on polydimethylsiloxane (PDMS) slides. Second, the slides are covered with a dialysis membrane and incubated with an optimized cell lysis solution containing 1% lithium dodecyl sulfate. Third, the mRNA binds to poly(dT) barcode beads, which are collected and subjected to reverse transcription to generate cDNA for next generation sequencing. (b) Total numbers of tags and genes measured for each unique barcode. (c) Scatter plots comparing the tag counts of randomly selected individual cells. When arbitrarily selected cells were compared, the gene expression patterns of most cells with higher sequenced reads were very similar (R^2^ = ~0.9), indicating high experimental precision. (d) The relative intensity of the housekeeping genes *GAPDH* and *B2M* for each unique barcode. In addition, the relative intensity of the housekeeping genes *GAPDH* and *B2M* was similar across all cells, including cells with lower reads. These results suggest that Nx1-Seq is a useful and valid way to characterize the gene expression of specific cell populations.
(TIF)

S2 Fig Expression and localization of hepatitis B core antigen (HBV-core; green) and the DENND2A and DOCK11 proteins (red) in HC1 cells. Representative images of three independent experiments. For indirect immunofluorescence analysis, a rabbit polyclonal anti-hepatitis core antigen antibody (anti-HBcAg; Thermo Fisher Scientific K.K., Kanagawa, Japan) was used. For immunostaining, cultured cells were fixed with methanol-acetone (1:1) for 10 min and permeabilized with 0.01% Triton X-100 (Merck, Darmstadt, Germany) in 10 mmol/L PBS (pH 7.5) for 10 min at room temperature. After further incubation in PBS containing 5% BSA for 30 min, cells were incubated overnight at 4°C with anti-HBcAg diluted in PBS containing 3% BSA. After washing in PBS containing Tween 20, cells were incubated for 1 h at room temperature with Alexa 488 donkey anti-goat and Alexa 594 donkey anti-rabbit secondary antibodies (Life Technologies Japan, Tokyo, Japan). Cell nuclei were stained with DAPI (Dojindo Laboratories, Kumamoto, Japan). To analyze the ratio of HBV-positive PHHs, five photographs were taken, and the number of PHHs and HBV-positive PHHs were counted.
(TIF)

S3 Fig Knockdown of DENND2A and DOCK11 by shRNA in HepG2.2.15 cells. On day 1, the lentiviral particle suspension was added to each well of a plate containing HepG2.2.15 cells. The cells (2 × 10^5^) were spread in 12-well plates to start the experiments. The plate was incubated overnight at 37°C. On day 2, the plates were washed with buffer. From days 3–6, the culture medium was replaced as necessary. On day 6, the cells were collected for the measurement of HBV content. The numbers (#) represent different constructs of shRNA. Data represent the mean ± SD pooled from three independent experiments. HBV DNA and cccDNA contents were compared to those of mock-transfected control cells. *P < 0.05; **P < 0.01; ***P < 0.001.
(TIFF)

S4 Fig cccDNA analysis. cccDNA levels were analyzed via Southern blotting after 14 days of shRNA treatment targeting DOCK11 in HBV-infected HepG2.2.15 cells showing HBV replication. To isolate cccDNA, the extracted DNA was treated with Plasmid safe DNase I as described in the Materials and methods section. Purified, non-denatured cccDNA was hybridized and supercoiled cccDNA bands were identified by their expected size (2.1 kb) and linearization upon EcoRI digestion (double strand linear; dslDNA) (3.2 kb). ① marker, ② purified cccDNA treated with mock control shRNA, ③ purified cccDNA treated with Dock11 shRNA, ④ purified cccDNA, ⑤ purified cccDNA digested with EcoRI.
(TIFF)

S5 Fig Long-term cell viability profile (1 month). Knockdown of DOCK11 did not result in cytotoxicity compared to the control. Cell viability was measured using the MTT assay.
(TIFF)

S6 Fig The CRISPR/Cas9 system for *DOCK11* reduced pgRNA and cccDNA in HepG2.2.15 cells. The cells were transfected with either *DOCK11* double nickase plasmid (catalog No. sc-406204-NIC) or mock control double nickase plasmid (catalog No. sc-437281-NIC) purchased from Santa Cruz Biotechnology (Santa Cruz, CA, USA) and then selected as *DOCK11* knockout (KO) or wild-type (WT) cells using culture medium containing puromycin. Briefly, cells were grown until 70% confluence on a 6-well tissue culture plate and then transfected with 3 μg of plasmid DNA using the Lipofectamine 2000 (Takara Bio). After 24 h of incubation, cells were selected with culture medium containing 5 μg/mL puromycin antibiotic (Sigma), resulting in the death of all non-transfected cells within 36 h.
(TIFF)

S1 Table shRNA sequence. (XLSX)

S2 Table Sequencing summary. (XLSX)

S3 Table Single cell gene expression profile in KM cells. (ZIP)

S4 Table Genes higly expressed in cells with HBV mRNA compared with control cells. (XLSX)

S1 Raw data (TIFF)
