# Supplementary figures and images for: DOCK11 and DENND2A play pivotal roles in the maintenance of hepatitis B virus in host cells
Source: PLoS One. 2021 Feb 4;16(2):e0246313. doi: 10.1371/journal.pone.0246313 (PMC7861363; doi:10.1371/journal.pone.0246313)

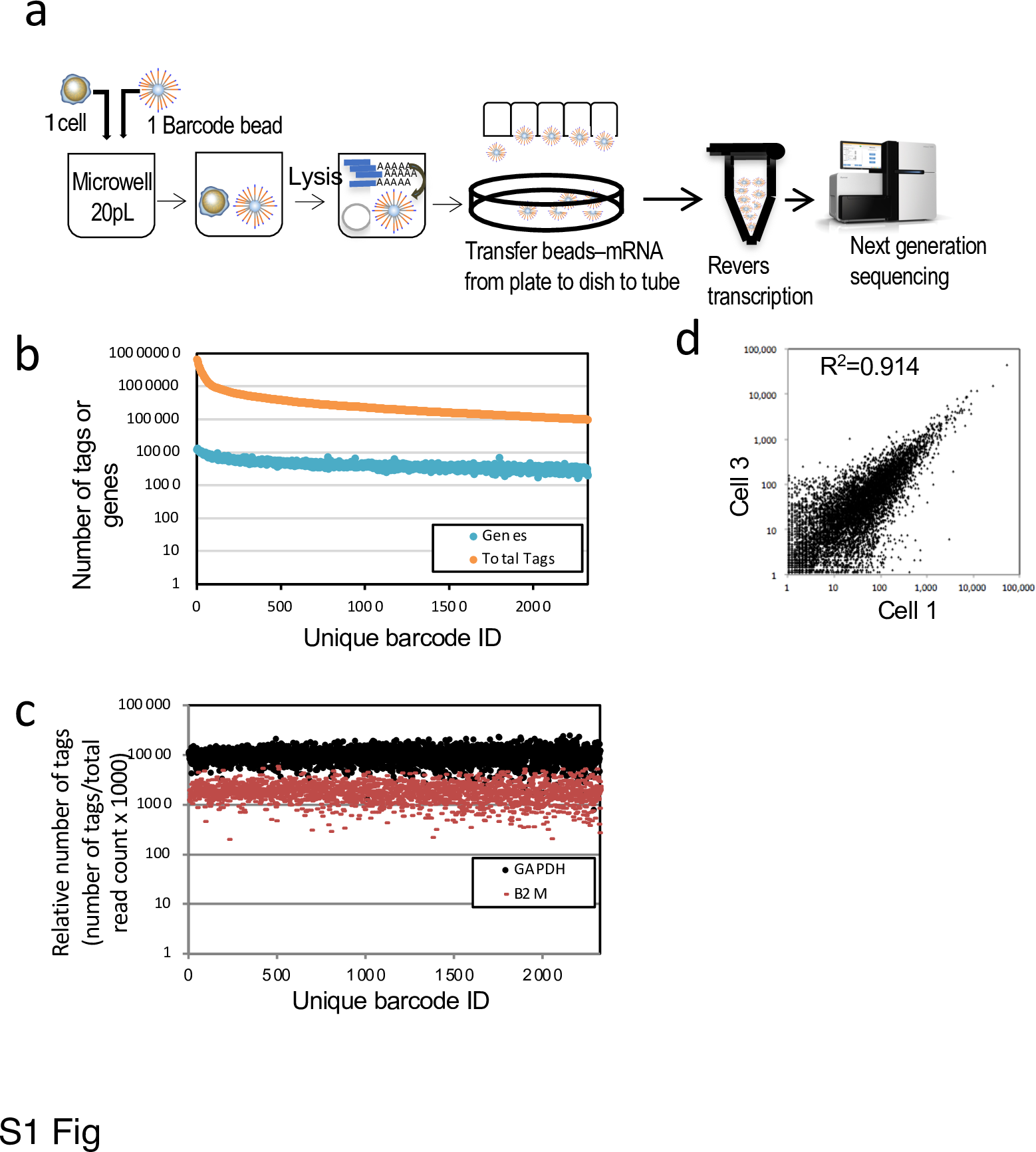

Supplement: S1 Fig — (a) Outline of the Nx1-Seq approach. First, single-cell and oligo-dT barcode beads are combined on polydimethylsiloxane (PDMS) slides. Second, the slides are covered with a dialysis membrane and incubated with an optimized cell lysis solution containing 1% lithium dodecyl sulfate. Third, the mRNA binds to poly(dT) barcode beads, which are collected and subjected to reverse transcription to generate cDNA for next generation sequencing. (b) Total numbers of tags and genes measured for each unique barcode. (c) Scatter plots comparing the tag counts of randomly selected individual cells. When arbitrarily selected cells were compared, the gene expression patterns of most cells with higher sequenced reads were very similar (R2 = ~0.9), indicating high experimental precision. (d) The relative intensity of the housekeeping genes GAPDH and B2M for each unique barcode. In addition, the relative intensity of the housekeeping genes GAPDH and B2M was similar across all cells, including cells with lower reads. These results suggest that Nx1-Seq is a useful and valid way to characterize the gene expression of specific cell populations. (TIF) [file pone.0246313.s001.tif]

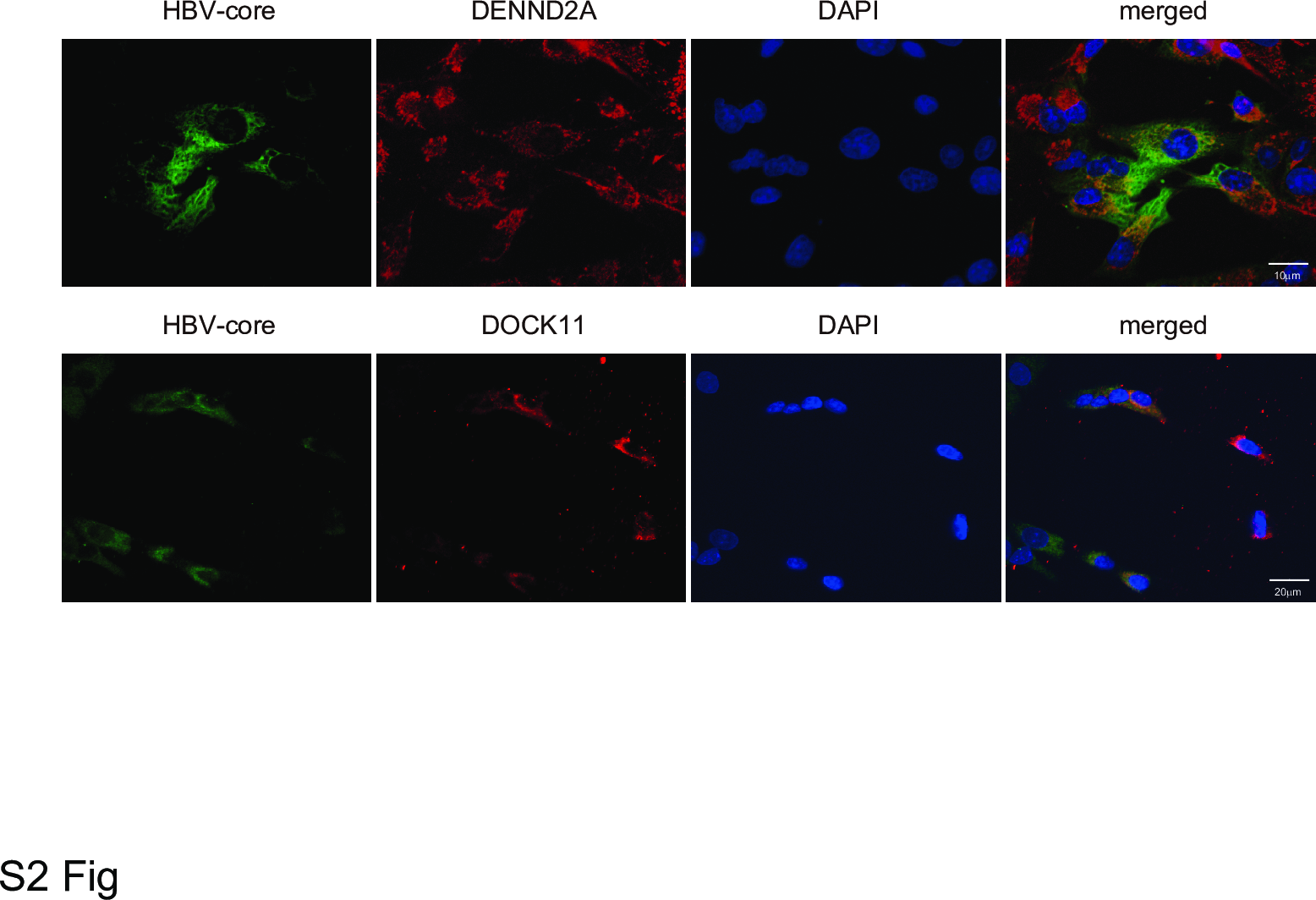

Supplement: S2 Fig — Representative images of three independent experiments. For indirect immunofluorescence analysis, a rabbit polyclonal anti-hepatitis core antigen antibody (anti-HBcAg; Thermo Fisher Scientific K.K., Kanagawa, Japan) was used. For immunostaining, cultured cells were fixed with methanol-acetone (1:1) for 10 min and permeabilized with 0.01% Triton X-100 (Merck, Darmstadt, Germany) in 10 mmol/L PBS (pH 7.5) for 10 min at room temperature. After further incubation in PBS containing 5% BSA for 30 min, cells were incubated overnight at 4°C with anti-HBcAg diluted in PBS containing 3% BSA. After washing in PBS containing Tween 20, cells were incubated for 1 h at room temperature with Alexa 488 donkey anti-goat and Alexa 594 donkey anti-rabbit secondary antibodies (Life Technologies Japan, Tokyo, Japan). Cell nuclei were stained with DAPI (Dojindo Laboratories, Kumamoto, Japan). To analyze the ratio of HBV-positive PHHs, five photographs were taken, and the number of PHHs and HBV-positive PHHs were counted. (TIF) [file pone.0246313.s002.tif]

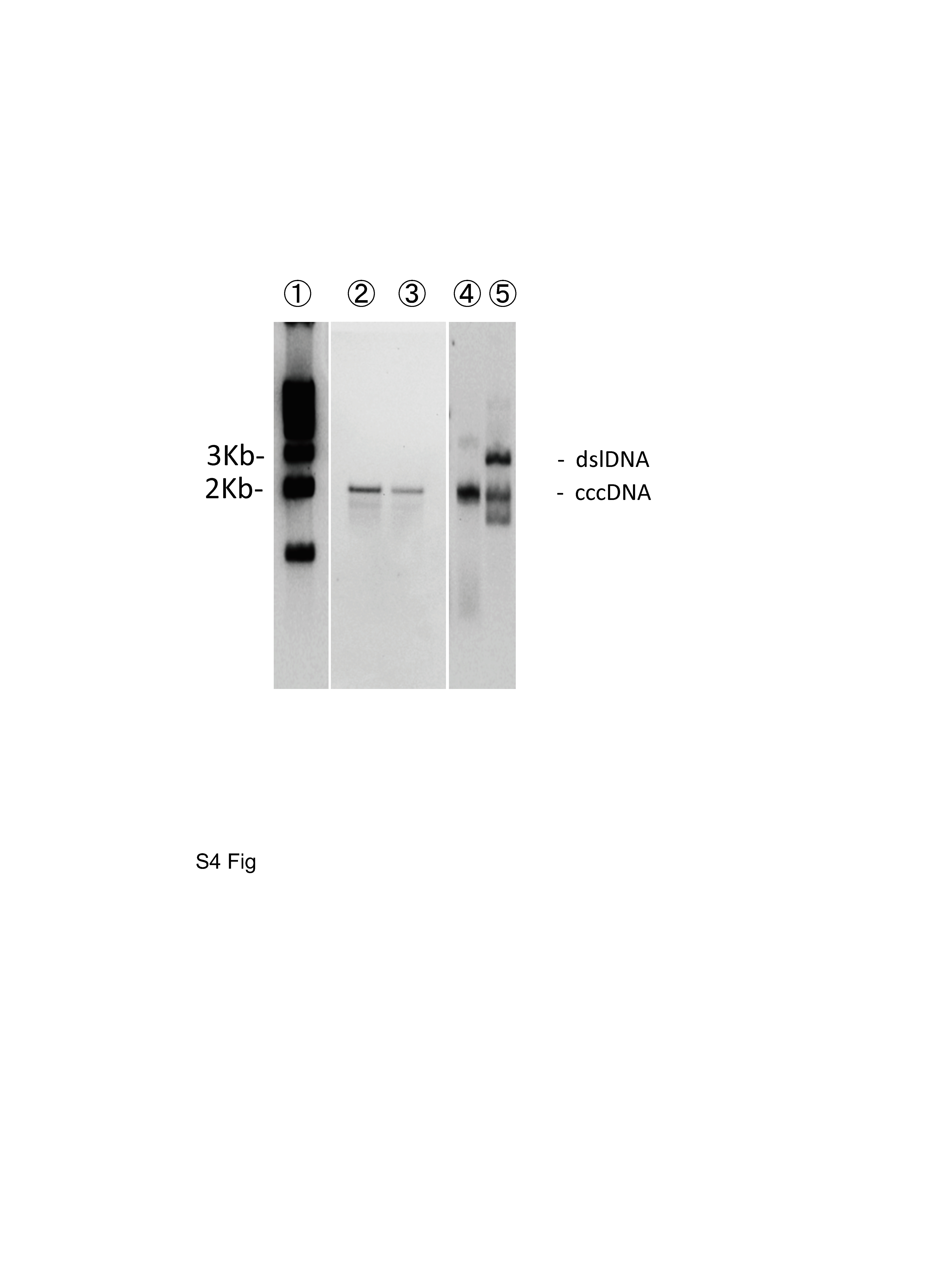

Supplement: S4 Fig — cccDNA levels were analyzed via Southern blotting after 14 days of shRNA treatment targeting DOCK11 in HBV-infected HepG2.2.15 cells showing HBV replication. To isolate cccDNA, the extracted DNA was treated with Plasmid safe DNase I as described in the Materials and methods section. Purified, non-denatured cccDNA was hybridized and supercoiled cccDNA bands were identified by their expected size (2.1 kb) and linearization upon EcoRI digestion (double strand linear; dslDNA) (3.2 kb). ① marker, ② purified cccDNA treated with mock control shRNA, ③ purified cccDNA treated with Dock11 shRNA, ④ purified cccDNA, ⑤ purified cccDNA digested with EcoRI. (TIFF) [file pone.0246313.s004.tiff]

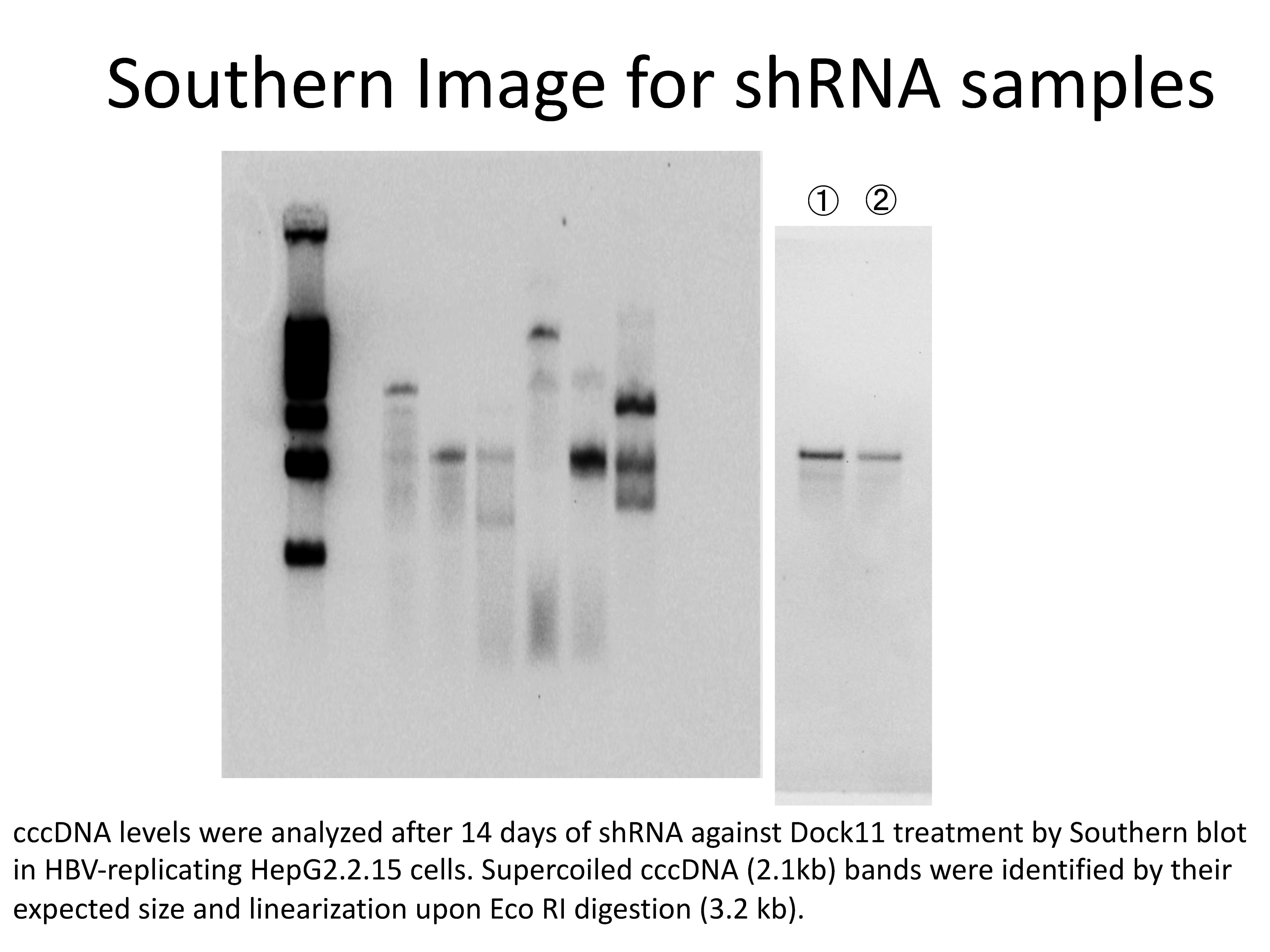

Supplement: S1 Raw data — (TIFF) [file pone.0246313.s011.tiff]
